# Investigation of the physical driving mechanisms of wind noise in hearing devices by computational fluid dynamics

**DOI:** 10.1038/s41598-025-93303-y

**Published:** 2025-03-25

**Authors:** Jörg Riedel, Stefan Becker, Christoph Näger, Felix Czwielong, Stefan Schoder

**Affiliations:** 1 Friedrich-Alexander-Universität Erlangen-Nürnberg, Institute for Fluid Mechanics (LSTM), Cauerstraße 4, 91058 Erlangen, Germany; 2 Graz University of Technology, Institute of Fundamentals and Theory of Electrical Engineering, Inffeldgasse 16c, 8010 Graz, Austria

**Keywords:** Hearing, Wind noise, Flow simulation, Flow-induced sound, Smartphone, Wearables, Hearing Aid, Biomedical engineering, Health care

## Abstract

Wind noise impairs the functionality of hearing aids and hearables outdoors or during sports by interfering with communication signals. This study aims to visualize the wind noise generation patterns around the human head by validated scale-resolved flow simulations. For the first time, the three-dimensional turbulent flow field at wind speeds of 10 km/h and 20 km/h around a female, a male and an artificial head is analyzed. It is possible to extract non-accessible data even inside the body, e.g., the pressure field deep inside the ear cavity in front of the eardrum. Head-geometry-independent flow features are identified. In the temple area, large-scale vortex shedding occurs. Small-scale vortices detach at the upper edge of the pinna and across the entire ear area. At typical microphone positions of behind the ear worn hearing devices, the pressure fluctuations are more pronounced than those at the auditory canal entrance. The tragus of the pinna plays a decisive role in attenuating wind noise in front of the entrance to the auditory canal. Anatomically exact ear canals ensure that velocity fluctuations are attenuated more effectively compared to an artificial one. At 20 km/h, the A-weighted pressure levels recorded at the microphone location of a behind the ear worn hearing devices exceed 85 dB(A). The results lead to a first understanding of wind noise effects and how they increase the perception threshold for recognition. Manufacturers can use the model to facilitate the wind noise optimal placement of microphones in new products to enhance communication under windy conditions.

## Introduction

The Centers for Disease Control and Prevention reported, in 2018, that 14% of Americans have some degree of hearing loss^1^. Among those aged 45 and over, 2.8% of men and 1.9% of women; aged 65 and over, 19.2% of men and 10.6% of women used a hearing aid^1^. Additionally, there are 6.9 billion smartphone users worldwide^2^. Both people with and without hearing loss suffer from wind-induced noise during communication outdoors.

**Fig. 1:**
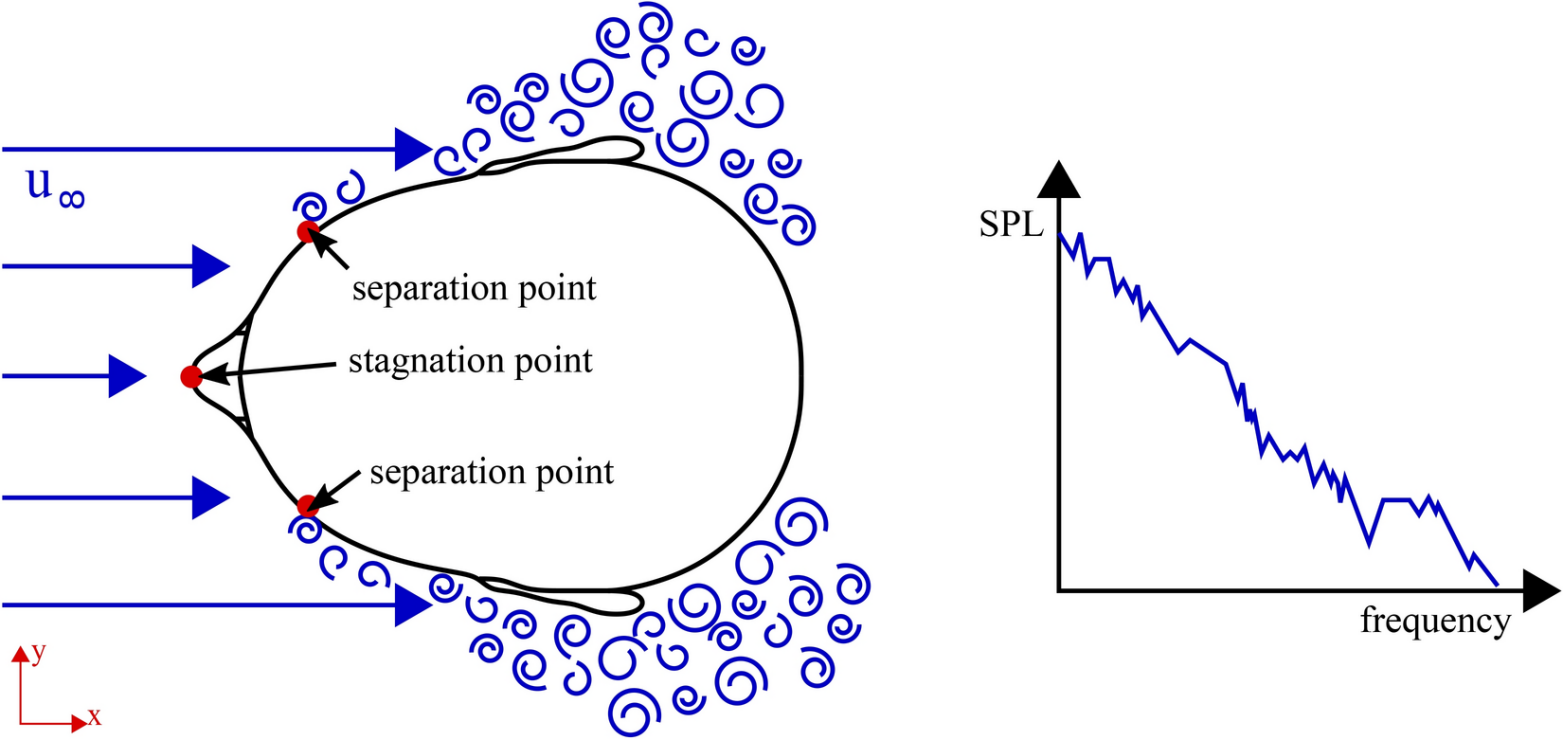
Visualization of flow features in the transversal plane of a human head which are masking sound waves noticed by the human ear. At the head exposed to frontal flow (blue), detachment occurs at the temple and in the area of the pinna. This results in a sound pressure signal (wind noise), which is particularly strong in the low-frequency range and decreases towards higher frequencies. The superimposition of the sound signal and the flow results in a masking of the useful sound signal by wind noise. Especially in the low-frequency region, the wind noise is masking the sound signal.

The acoustic signal during communication is perceived as acoustic pressure waves (pressure fluctuation) at the ear, whereas wind-induced noise is an additional (incompressible) pressure fluctuation around the ear driven by the geometrical shape of the head. Figure 1 summarizes schematically the previously reported flow effects^3–7^, like stagnation and flow separation at the cheeks, in the transverse plane of the human head. For the perceived pressure signal at typical wind loading, the incompressible pressure fluctuations of the wind $p_w$ dominate the recognized signal at low frequencies, generally being perceived as broadband turbulent rumbling^8^. It is this characteristic sound of wind everyone knows; it can cause unpleasant saturation in hearing devices. From a physical point of view, the acoustic pressure waves $p_a$ and the wind-induced noise $p_w$ are two components of the overall pressure fluctuation $p' = p_a + p_w$ perceived during communication^9^.

From a communication perspective, signal levels at distinct signal frequencies are important. The sound signal $p_a$ carries the information from the speaker to the listener, whereas $p_w$ is sometimes referred to as noise in signal processing^4^, masking the communication signal to some extent. Clear communication is given when the signal-to-noise ratio is high at a given signal frequency. Therefore, understanding the correlation between wind-induced sound levels $p_w$ at the human ear and wind speed in relation to the threshold for recognizing audible signals $p_a$ is important.

**Fig. 2:**
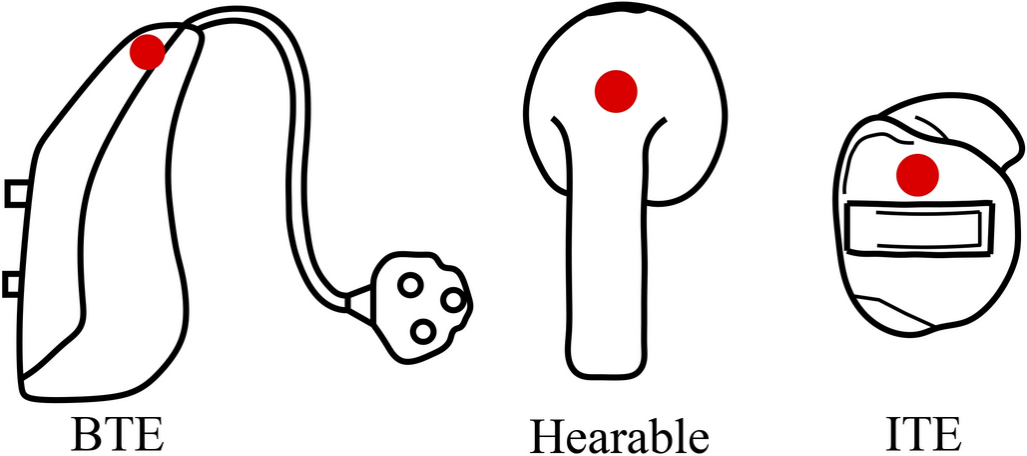
Schematic drawing of different types of hearing devices, BTE device (left), Hearable (centre) and ITE device (right) with the marking of possible microphone positions for the recording of ambient sound (red circles).

Figure 2 provides a schematic overview of the designs of the different hearing devices described, including possible microphone positions. The classic and today’s most widely used hearing aid is the BTE (Behind The Ear) device^10,11^. This device contains microphones and loudspeakers in a banana-shaped housing (see Fig 2 BTE). The housing is worn behind the pinna and the sound is transmitted acoustically to the ear canal via a plastic tube. Some devices are worn in and on the pinna (see Fig 2 ITE). Within this group, the ITE (In The Ear) devices^10^ usually fill the entire pinna of the patient’s ear with their housing. The ITC (In The Canal) devices^10^ have a surface that is flush with the entrance to the patient’s ear canal and are, therefore, smaller than the ITE devices. Finally, the CIC (Completely In the Canal) devices^10^ disappear entirely inside the patient’s ear canal and are barely visible from the outside. The collective term hearables refers to small smart devices worn in the ear canal that have both microphones and speakers (see Fig 2 hearable). They are able to connect to a smartphone or the internet wirelessly^12^ and are characterized by advanced software-based functions such as active noise canceling or augmented reality^13^. They are a low-barrier option for people with mild to moderate hearing loss to equip themselves with hearing support devices^14^. There are also efforts to use such devices for extended health monitoring and to record cardiac rhythm^15^ or brain and respiratory functions^16^, for example.

Different advances in understanding the physics of wind-induced noise effects in hearing aids have been made using experimental studies^3–7^. Daalsgard et al.^3^ carried out tests in the wind tunnel on five BTE hearing aid variants with modified microphone ports at wind speeds of 7 m/s. They found that the wind noise can be attenuated by protective devices directly on the microphone port and by changing the microphone position. However, the authors found that the pressure fluctuations recorded by the hearing aid depend to a large extent on the head itself and the geometry of the pinna, meaning that the design of the BTE device has a limited influence. Fortune and Preves^4^ compared the wind noise performance of ITE, ITC and CIC devices at a wind speed of approximately 3 m/s. The detected noise level was lowest for the CIC device and highest for the ITE device. It was deduced as a rule of thumb: the deeper the hearing aid is positioned in the ear canal, the better the performance of the hearing aid on average.

Chung et al.^7^ studied the polar wind noise attenuation characteristics of BTE devices in an aeroacoustic wind tunnel using the artificial head KEMAR^17^. Wind speeds of 0 m/s, 4.5 m/s, 9.0 m/s and 13.5 m/s were used. It was found that the highest sound pressure levels at the BTE device microphone position are achieved when the head is exposed to the airflow completely from the front (0$^\circ$) or completely from behind (180$^\circ$). If the ear with the BTE device is pointing directly into the approaching wind (90$^\circ$), the measured sound pressure levels decrease drastically. When pointing directly into the wind, for a wind speed of 4.5 m/s a reduction of 30 dB is achieved in the sound pressure level averaged over the entire frequency range. The authors explain this physically by the fact that the flow velocity at the stagnation point is theoretically zero.

Zakis^6^ carried out tests with different hearing aids in the wind tunnel at 3, 6 and 12 m/s. He examined the recorded pressure spectra for two BTE hearing aid variants and a CIC hearing aid worn by KEMAR. The two BTE devices were clearly inferior to the CIC device in terms of wind noise under frontal flow conditions supporting the findings by Fortune and Preves^4^. At the lowest wind speed of 3 m/s, the sound pressure level at 200 Hz of the BTE devices was 10 dB higher than that of the CIC device. At 1000 Hz, the difference was 30 dB. It was shown experimentally that the differences in detected sound pressure levels between the hearing aids become smaller with increasing the wind speed. At 12 m/s, the sound pressure level difference at 1000 Hz was only 8 dB. While for a wind speed of 3 m/s the microphone of the BTE device showed an increased sound pressure level in the range between 100 Hz and 2000 Hz compared to the quiescent measurement, at a speed of 12 m/s the entire frequency range under consideration between 100 Hz and 10000 Hz was affected.

Dillon et. al.^5^ carried out experiments at a wind speed of 5 m/s in a wind tunnel using KEMAR^17^. BTE, ITE, ITC and CIC hearing aids were examined. The three components of the flow velocity were determined using LDA (Laser Doppler Anemometry). The pressure spectra were recorded at the microphone positions. It was found that the turbulent velocity fluctuations decrease rapidly with increasing distance from the head. At a distance of 10 mm the turbulent fluctuation velocity equals 2 m/s and at a distance of 20 mm it has already decreased to 0.5 m/s. It was also shown that the strongest wind noise occurs when the BTE device, which is worn behind the ear, is approached by the wind from the front. The BTE device detected a sound pressure level over the entire frequency range of interest of 100-5000 Hz that was at least 10 dB higher than that of the other devices. From an experimental viewpoint, the following qualitative statements are synthesized by the literature review:

- The very low-frequency components in the spectrum are related to the head geometry, in the mid-frequencies, the shape of the pinna is an essential factor, whereas the high-frequency component in the spectrum is a result of the tragus and microphone port geometry^5^.
- As the wind speed increases, the wind noise pressure signal level rises and the frequency range of occurrence in which the disturbances are detected is extended^6^.
- The wind noise level is lowest where the microphone points directly into the approaching wind^7^.
- The deeper the hearing device is positioned in the ear canal, the better the performance of the hearing aid on average^4^.

To conclude, there are already several qualitative findings published in the relevant literature. However, there is a significant gap in understanding the physics of the pressure fluctuations related to primary flow field features around the human head. The main objective is to use for the first time high-fidelity Computational Fluid Dynamics and Aeroacoustics theory to resolve the flow structures of wind noise around the human head. We visualize these structures to gain deeper and quantitative insight into the physical effects of wind noise (Fig. 1), reported as qualitative statements in previous literature^3–7^. The simulation models derived are useful to improve medical devices for hearing loss compensation and communication in windy environments and enhance the overall user experience of smartphone users. It contributes to the life quality of people with substantial hearing loss^18,19^ and positively impacts a large proportion of society using communication devices outdoors.

## Flow configuration

**Fig. 3:**
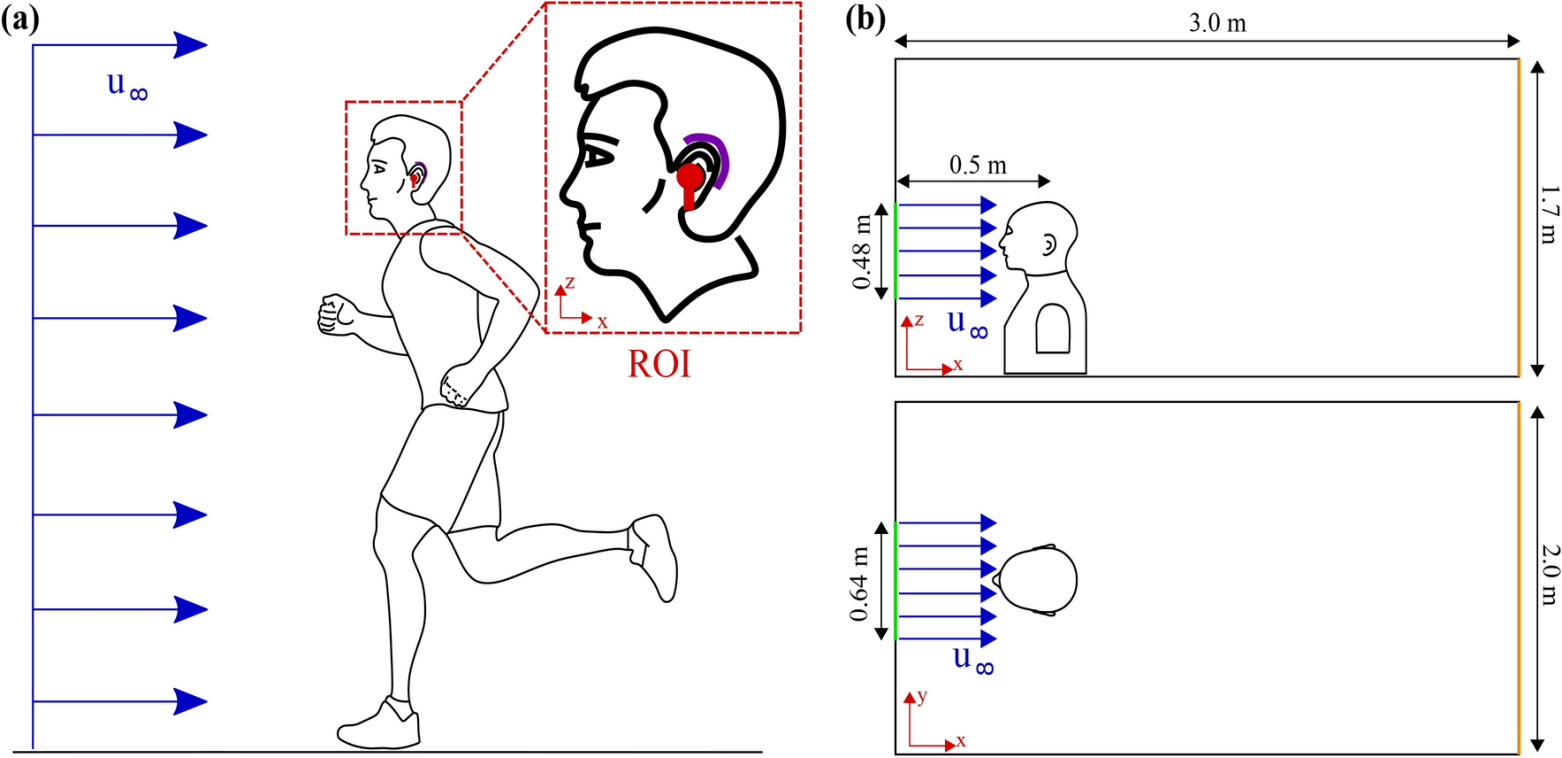
(**a**) Exemplary scene for the wind noise problem analyzed in this study. A jogger is running at a speed of 10 km/h. Due to the relative speed to the quiescent air ($u_\infty$), he is exposed to a flow profile (shown in blue). The region of interest for the simulation (ROI) is shown again in detail. The runner wears either a hearable located in the ear canal (red) or Behind The Ear hearing aids (purple). (**b**) Sketch of the flow domain in the flow analysis. The head model is positioned at a distance of 0.5 m from the outlet of a wind tunnel nozzle with a cross-section of 0.64 m x 0.48 m. The domain has a total length of 3.0 m, a height of 1.7 m and a width of 2.0 m. The simulation inlet boundary condition is highlighted in green and the outlet boundary condition in orange.

There are different driving mechanisms for wind noise. A first variant is meteorological wind noise - gusts of wind that arise on the beach, for example, approaching the person using a hearing aid or a hearable. The first variant is less suitable for the fundamental investigations intended in this paper, i.e. the occurrence of strongly transient flow effects coupled with vortices of widely varying sizes transported by the gust. A second variant is the movement of a person in quiescent air, whether by walking, jogging, inline-skating or cycling, where the relative speed of the moving person also causes wind noise. We consider jogging or cycling that can be replicated in an aeroacoustic wind tunnel, as shown in Fig. 3a. Typical wind speeds investigated in previous experimental studies are in line with the two investigated speeds of 10 km/h and 20 km/h.

In Fig. 3a, a jogger moves without any other external wind influences. The subject is wearing either a hearable (shown in red) or a behind-the-ear hearing aid (shown in purple). Due to the speed relative motion of the jogger to the air and ground, the jogger is exposed to a primary airflow indicated by the velocity $u_\infty$, as shown in the blue profile in the sketch. At the level of the navel, a uniform flow can be approximately assumed. Secondary flow effects due to up and down movement from the subject while running are neglected. The situation is similar to rolling down a hill with inline skates or a bicycle. Figure 3b illustrates the geometrical situation of the validation experiment in the wind tunnel and the corresponding validated flow simulation, where further details on the methods are provided in Section Methods.

## Methods

The experiment in the wind tunnel and the corresponding validated Computational Fluid Dynamics (CFD) simulation follows the dimensions of Fig. 3b. At a distance of 0.5 m from the wind tunnel nozzle the model of the person geometry under investigation is positioned. In the front of the model of the person in a cross-section of 0.64 m x 0.48 m a uniform flow profile with a degree of turbulence of less than 0.5 % was measured via Hot Wire Anemometry, ensuring conditions of low turbulence inflow being a typical value for case of moving bodies in quiescent air. The main flow is modeled along the x-axis of the Cartesian coordinate system fixed with the human body. Each head model extends from the crown to approximately the navel. There is sufficient space in the simulation domain in the height and width direction to correctly reproduce displacement effects and detachment phenomena to the side and behind the head.

### Head geometries

**Fig. 4:**
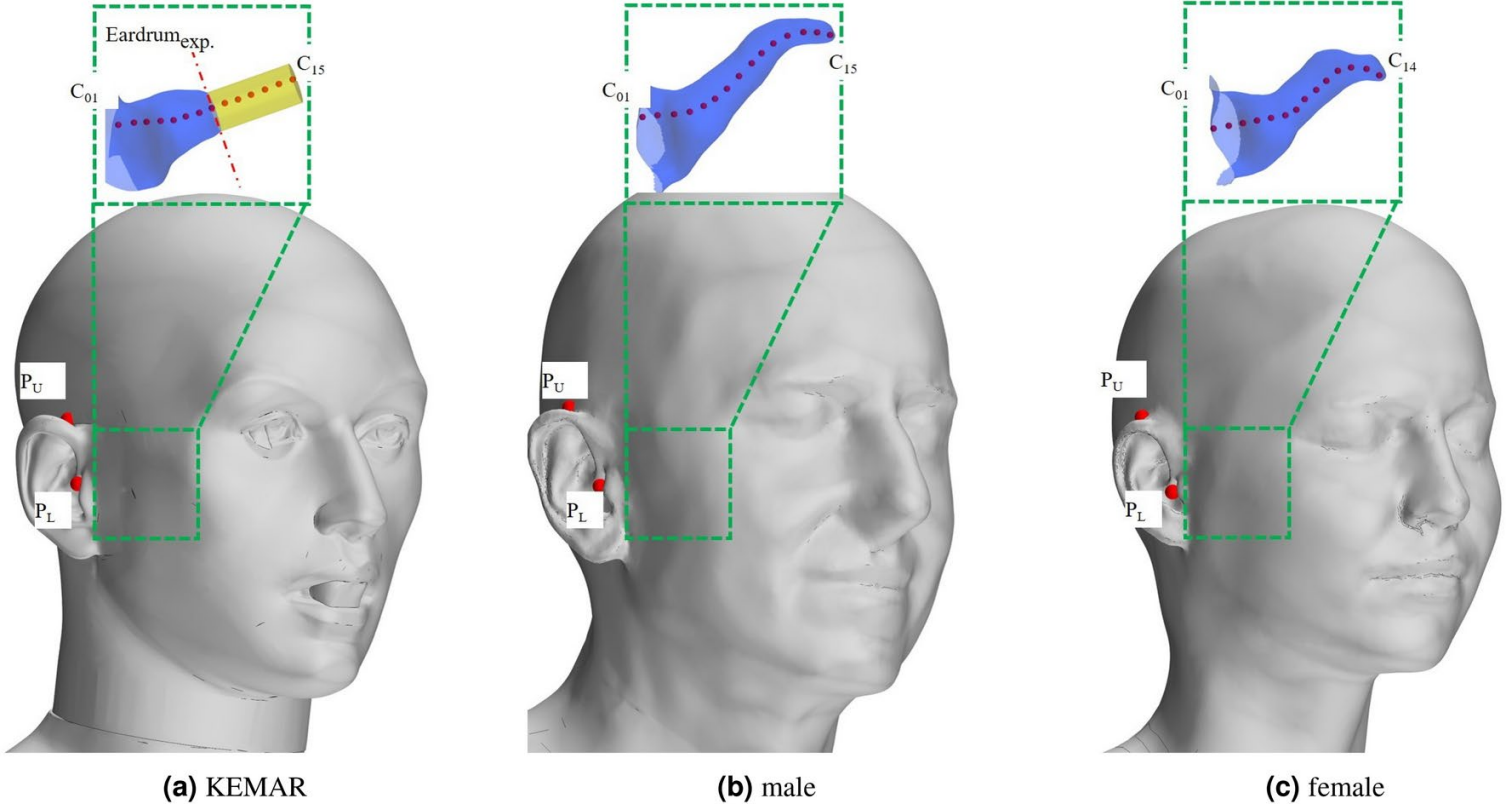
Close-up view of the analysed head models. The monitor points used in the analyses are shown in red. Here, $P_{L}$ denotes the monitor point in the lower reference plane in front of the entrance to the auditory canal; $P_{U}$ denotes the monitor point in the upper reference plane, at the position of a hearing aid microphone. The enlarged zoom view shows the auditory canals inside the heads. Points $C_{01}$ to $C_{15}$ describe points inside the ear canal. In the case of the KEMAR, the original, shorter auditory canal (blue) was extended with the help of an extrusion (yellow) for better comparability. The red dashed line indicates the position of the microphone in the experiment.

Figure 4 shows a close-up of the CAD models of the head and torso (not shown) geometries analyzed in this study.

The CAD model of the artificial head KEMAR from the company G.R.A.S is shown in subfigure 4a. The physical version of the artificial head KEMAR geometry is equipped with microphones. It was analyzed in the wind tunnel and the experimental results serve as a validation reference in this study. As part of this work, we test to what extent the artificial head model KEMAR, which is the standard manikin for acoustical measurements in the hearing aid industry^20^ realistically reproduces the fluid dynamic behavior compared to two real humans, one female and one male. The other two head models of the real people correspond to 3D scans of a female and a male from the IHA database^21^. Subfigure 4b shows the head of a man with a height of 1.84 m and a weight of 75 kg. Subfigure 4c represents the head of a woman with a height of 1.70 m and a weight of 57 kg. As part of our study, wind tunnel tests were carried out exclusively on an artificial head KEMAR. No human subjects were used for the experimental wind tunnel tests. The two human heads were only examined numerically. The CAD data used for this computational study was obtained from other researchers through scans. These scans are available in a public database^21^. Informed consent was obtained from all subjects of this study and the respective CAD data were treated in accordance with the relevant ethical standards.

In order to monitor the velocity fluctuations and pressure fluctuations during the simulation, we define several monitor points for all heads (without placing the actual device), as shown in Fig. 4. The monitor point $P_{L}$ (Lower) is located on a plane at half the height of the entrance to the auditory canal. The microphones of a hearable or an ITE-device would be located at approximately this point. The monitor point $P_{U}$ (Upper) is localized in a plane that intersects the center of the zygomatic bone and is located where the front microphone of a BTE hearing aid is assumed to be. In addition to the points located outside the head, we define further monitor points inside the ear canal to check how quickly the flow-induced pressure and velocity fluctuations in the ear canal dissipate. The definition of these points can be seen in the zoomed-in sections in Fig. 4. Here, point $C_{01}$ is the point directly adjacent to the entrance to the ear canal and is located in a total distance of 2.5 mm from $P_{L}$. The other points are then located further inside the ear canal at a distance of 2.5 mm each. For the two heads from the IHA database (man and woman), a physiological geometry of the ear canal is available, which was obtained by an MRI scan and is shown in blue in the enlarged images. For the artificial head KEMAR, only a shorter anthropometric auditory canal is available for both the measurement manikin and the CAD model, which is also colored blue. The microphone membrane of the manikin is located at the end of the shortened ear canal, which is indicated in the drawing by the red dotted line. The reference surface for the validation simulation is also located here, see Fig. 6. For comparison purposes and to match the length of the physiological ear canals of the other two heads, the auditory canal of the KEMAR was additionally extended in the form of a simple cylindrical geometry for a further simulation (yellow part of the auditory canal in subfigure a).

### Flow model

We use the commercial flow solver STAR-CCM+ (17.02.007) for the CFD investigations solving the incompressible Navier-Stokes equations. The domain is meshed with a trimmed-cell mesh, which consists of cube-shaped elements. We resolve the boundary layer in the head region by applying 18 layers of prismatic cells. In total, each configuration consists of approximately 80 million grid cells.

A converged steady-state Reynolds-averaged Navier Stokes simulation with 2000 iterations based on the k-$\omega$-SST model calculates the initial solution for the subsequent transient CFD simulation. We are conducting a wall-resolved large eddy simulation (LES) with the WALE subgrid model for the unsteady flow simulation. Averaged over the entire head surfaces, we achieve values of $\Delta y^{+} = 0.37$, and $\Delta x^{+} = \Delta z^{+} = 10.6$ for the speed of 20 km/h and values of $\Delta y^{+} = 0.2$ and $\Delta x^{+} = \Delta z^{+} = 5.86$ for the speed of 10 km/h with the calculation grid used. Therefore the grid is fine enough to do fully wall-resolved LES, which requires $\Delta x^{+} < 100$ and $\Delta z^{+} < 20$^23^. From a purely grid perspective, we even meet the criteria for direct numerical simulation (DNS) which are given as $\Delta x^{+} < 20$ and $\Delta z^{+} < 10$^24^. There should be at least 40 grid nodes per resolved wavelength^25^ to resolve the pressure inside the ear. It requires a grid size of $\Delta x<c/(40f)=3.4$ mm in the ear canal to cover a frequency range of up to 2500 Hz (where *c* stands for the speed of sound). The mesh meets this condition, as the cube-shaped cells inside the auditory canal of the computational grid have a size of 0.3 mm (a factor of 10 finer than necessary).

The calculation method is based on the finite volume method. For convection a hybrid 3rd-order MUSCL/CD scheme was used, which is predestined for highly turbulent flows, especially when aero-acoustics are included^26^. The scheme exhibits reduced numerical dissipation compared to purely second-order methods and provides more accurate results for a variety of transient problems than lower-order methods^27^. Pressure-velocity coupling and the time evolution is based on a second-order Simple algorithm. Double precision was selected for the number format. A time step of 50 $\mathrm {\mu }$s, which corresponds to a sampling frequency of 20,000 Hz, is chosen. A total of 0.5 s of physical time is simulated per configuration; the transient effects of the initial conditions settled within the first 0.2 s of the simulation to a fully developed flow field. For the evaluation of the pressure spectra and velocity fluctuations, the time range between 0.2 s and 0.5 s was considered. The wind tunnel nozzle is modeled with a velocity inlet (see green line in Fig. 3), the side walls, the floor and the ceiling of the simulation domain are modeled with a symmetry boundary condition. A pressure outlet (see orange line in Fig. 3) is located at the rear end of the CFD domain, through which the flow leaves the domain. The boundary condition at the surface of the head model is described by a no-slip, rigid wall. These boundary conditions were used for the converged steady-state Reynolds-averaged Navier Stokes simulation and the subsequent time-dependent large eddy simulation. The boundaries are set far enough from the region of interest that they do not disturb the flow field. All simulations were carried out on the parallel CPU cluster *Fritz* of the National High Performance Computing Center Erlangen. Sixteen nodes were used per job. One node consists of 2 x Intel Xeon Platinum 8360 (Ice Lake), 2 x 36 cores with a clock frequency of 2.4 GHz^28^.

**Fig. 5:**
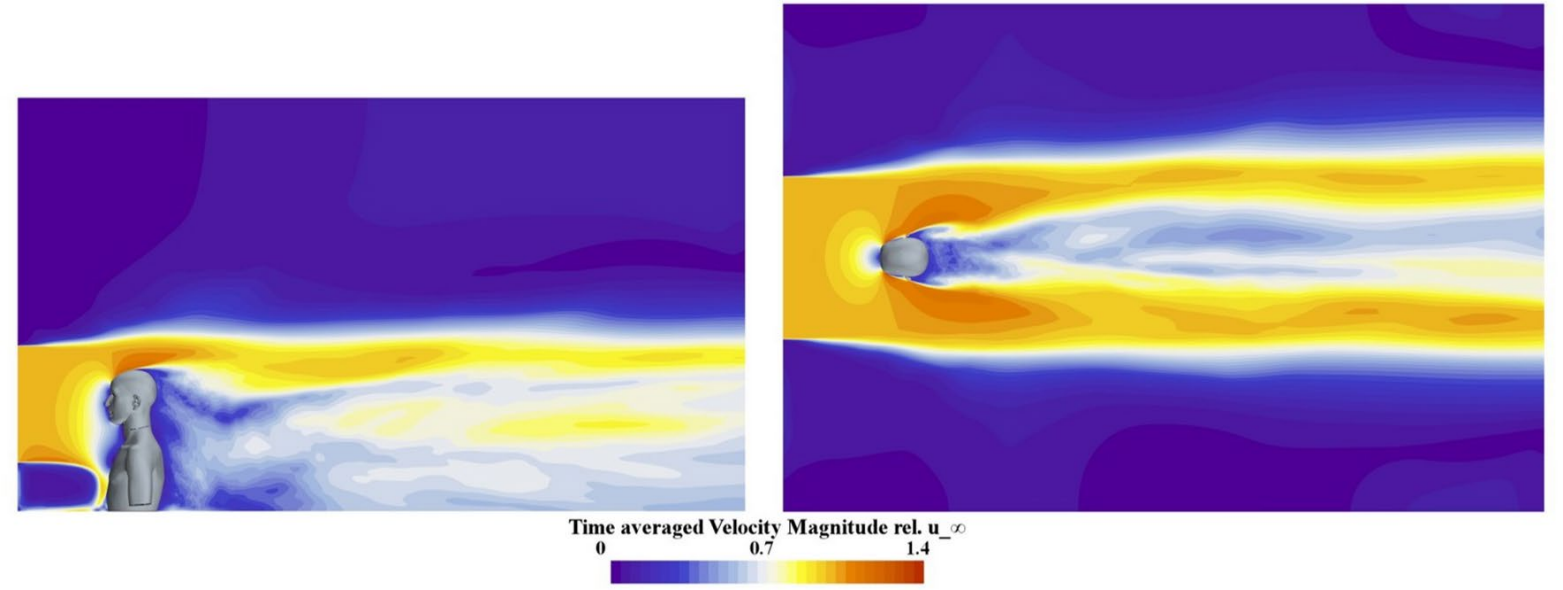
Representation of the free-stream normalized time-averaged flow velocity magnitude in the x-z plane on the left and in the x-y plane on the right for a inflow velocity of 20 km/h.

In the investigations, we consider two different flow velocities from possible everyday activities. On the one hand, 10 km/h corresponds to a typical average jogging speed, and on the other hand, 20 km/h is a typical average speed for relaxed cycling. Figure 5 provides a general overview of the flow field in the calculation domain. The figure shows the free-stream normalized time-averaged magnitude of the flow velocity around the KEMAR at a speed of 20 km/h in the planes according to Fig. 3b. The illustration shows that the calculation domain is large enough in all spatial directions and that artificial effects at the outer boundary conditions do not distort the results.

If we abstract the present fluid mechanics problem to the flow around a sphere and calculate with the equivalent head diameter of 0.021 m as the characteristic length, we achieve a Reynolds number of $Re_{D}=74,000$ for the 20 km/h and $Re_{D} =37,000$ for the 10 km/h. If one follows the contour of the KEMAR from the tip of the nose to the evaluation point $P_{U}$, the unwound length is 0.016 m. If the Reynolds number is formed using the distance travelled by the flow along the head surface, the resulting values are $Re_{x}=57,000$ for the 20 km/h and $Re_{x}=27,500$ for the 10 km/h. Ambient conditions of 25$^\circ$ Celsius are chosen for the material parameters of the air.

### Experimental validation

**Fig. 6:**
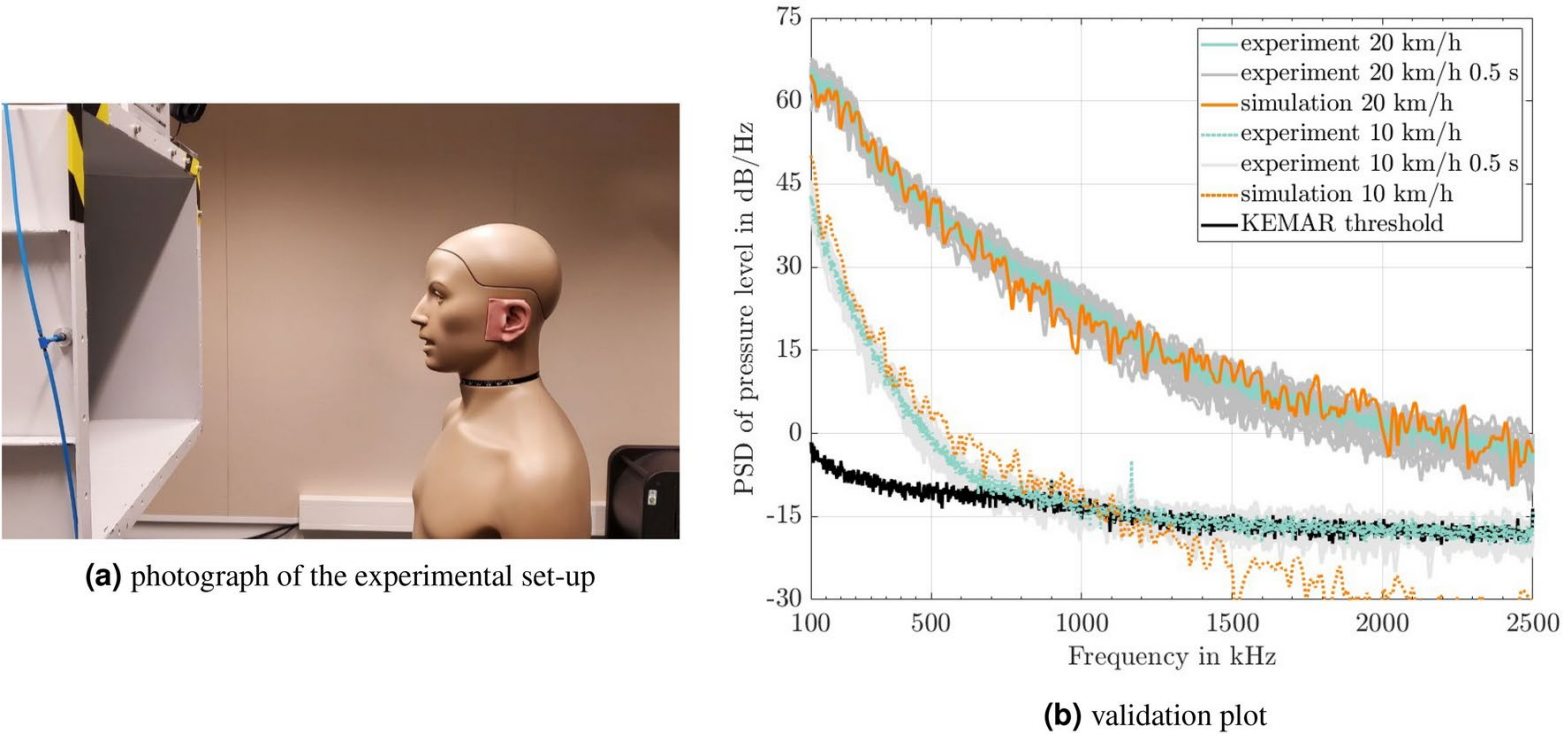
Picture of the experiment inside the aeraocoustic wind tunnel (**a**) and validation-plot for the simulative approach for the two wind velocities considered in this work, 10 km/h and 20 km/h, (**b**). Plotted are the power spectral densities (PSD) of the sound pressure levels over frequency at KEMAR’s right Eardrum. Plotted in black is the measurement threshold of the artifical head. Depicted in green is the averaged result of the measurements for 15 s measurement time. The grey set of curves represents the measurement results for a measurement time according to the simulation time of 0.5 s. Displayed in orange are the results for the CFD-simulations.

Before looking more closely at the qualitative description of the flow field around the heads, the simulation approach is first validated by wind tunnel experiments. A photograph of this experimental set-up is given in Fig. 6a. The experiments were carried out in an aeroacoustic wind tunnel with a low background noise level. The quiet supply of airflow is realized with the help of splitter silencers upstream and downstream of the fan. The measuring chamber is fitted with sound-absorbing wall paneling. At the wind speeds of 10 km/h and 20 km/h considered, the degree of turbulence of the flow at the nozzle outlet is less than 0.5 % across the entire nozzle cross-section. A KEMAR 45BB-10 with anthropometric ears from G.R.A.S. was used as the artificial head in the experiments. The artificial head is equipped with a microphone and a connected coupler inside the ear canal as standard. This position of the microphone at the end of the ear canal enables the pressure fluctuations to be measured inside the ear, similar to how sound is recognized by a human ear. Data was recorded at a sampling frequency of 48,000 Hz using the SQuadriga II mobile audio interface from HEAD acoustics. The measurement time was 15 seconds per measurement campaign.

Figure 6 shows plots of the power spectral densities of the sound pressure levels in the ear canal versus frequency for the wind speeds of 10 km/h and 20 km/h. Firstly, the black line represents the lower signal threshold of the artificial head. This is the minimum sound pressure level that can be recorded by the measurement equipment when the wind tunnel is switched off. Any acoustic signal below that black curve cannot be distinguished from the noise in the measured signal. The green curves are the averaged measurement results for the entire measurement time of 15 s. Printed in gray are the sets of curves for measurement intervals of 0.5 seconds each. This 0.5 s period corresponds to the total time period considered in the CFD simulation. The gray envelope represents the variability in the measured signal over the measurement time. Finally, the flow simulation results are shown in orange. The comparison of the experimental data with the simulation results shows good agreement over the considered frequency range of 100-2500 Hz. The pressure spectra are well-matched for both velocities analyzed. For the 20 km/h, excellent agreement of the simulation and the measurements is obtained over the whole considered frequency range. Some minor deviations within the gray envelope are present around 900 Hz. The decay of the simulated spectrum over the whole frequency range matches the experimental ones. The extended range of the gray curves at 20 km/h underlines the highly unstationary flow field characteristics.

For the 10 km/h, good agreement with the measurement results is obtained below 1200 Hz. Above that frequency, the deviations between the measured and simulated results can be explained by the noise threshold of -15 dB/Hz of the experiments. Physically, a further decay as seen in the simulation results is expected by the turbulent energy cascade^29^. From the validation, it can be concluded that the large eddy simulation resolves the necessary turbulent scales for the frequency range of 100-2500 Hz (20 km/h) and the frequency range of 100-1500 Hz (10 km/h) realistically such that the turbulent pressure fluctuations at the ear are calculated correctly. Validated by measurements, the flow simulation model can be combined with CT scans, MRI, or other imaging methods and can provide patient-specific analysis and assist tailored treatment^30^.

## Results

The value of the computational fluid dynamics simulation is particularly driven by making the invisible flow structures visible^31–37^. In addition, we can obtain quantitative information of experimentally inaccessible quantities such as force distribution^38^, energy^39^, the streamlines, flow paths and destination of particles^34^, origins of vortical structures, pressure field distribution^31^ and make them accessible for the human understanding and reasoning.

**Fig. 7:**
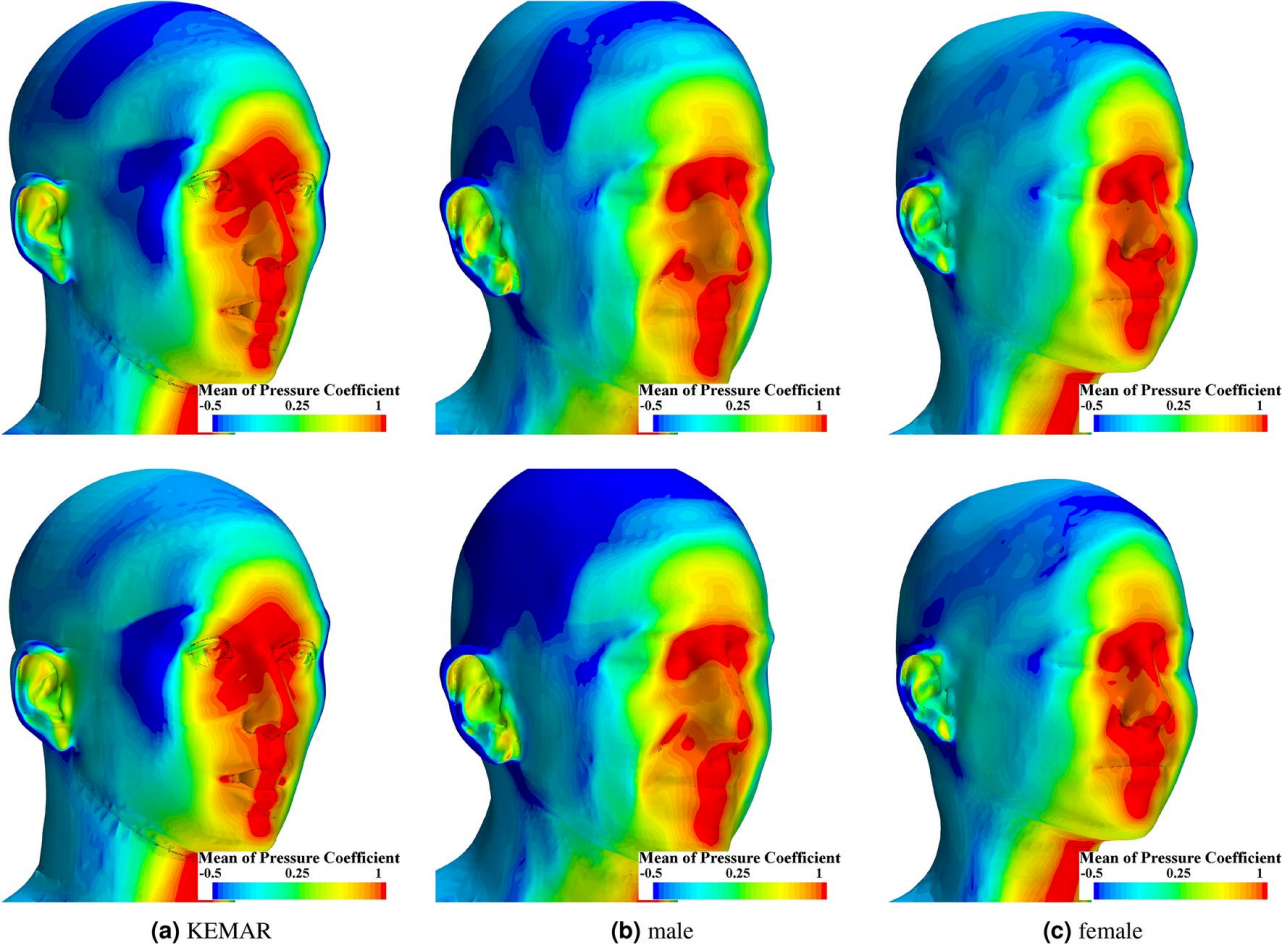
Time averaged pressure coefficient $\overline{c_{p}}$ for the three different head geometries at a wind velocity of 10 km/h (upper row) and 20 km/h (lower row).

In the case of the flow around bluff bodies, the distribution of the pressure coefficient $c_p = \frac{p - p_\infty }{ \frac{1}{2} \rho _\infty u_{\infty }^2 }$ and the skin friction coefficient $c_f = \frac{\tau _w}{ \frac{1}{2} \rho _\infty u_{\infty }^2 }$ are used for qualitative characterization of flow features. *p* denotes the pressure, $p_\infty$ the free-stream pressure, $\rho _\infty$ the free-stream density and $\tau _w$ the skin friction. Figure 7 first shows the time-averaged distribution of $c_p$ on the surfaces of the head models for a wind speed of 10 km/h in the upper row and a wind speed of 20 km/h in the lower row.

A maximum in the pressure coefficient indicates stagnation. When the flow approaches the head centrally from the front, it is located along the sagittal plane for each head geometry and wind velocity. At the stagnation, the kinetic fluid energy is completely converted into a pressure rise. In the case of a negative pressure coefficient, the flow is accelerated relative to the incident flow velocity. This is the case for the KEMAR over a large area in the zygomatic and sphenoid bone regions. The acceleration in this area is much less pronounced for the female and male due to the less pronounced geometrical details but still noticeable. In these cases, the acceleration is limited to the area of the temple. Common to all three heads is the negative pressure coefficient in the area of the crown of the head geometry. A comparison of the two wind speeds shows that the distribution of the pressure coefficient is very similar in both cases. Only at the back of the skull it is noticeable that the pressure coefficient for the male and female head geometry is decreased.

### Vortex formation and flow separation

**Fig. 8:**
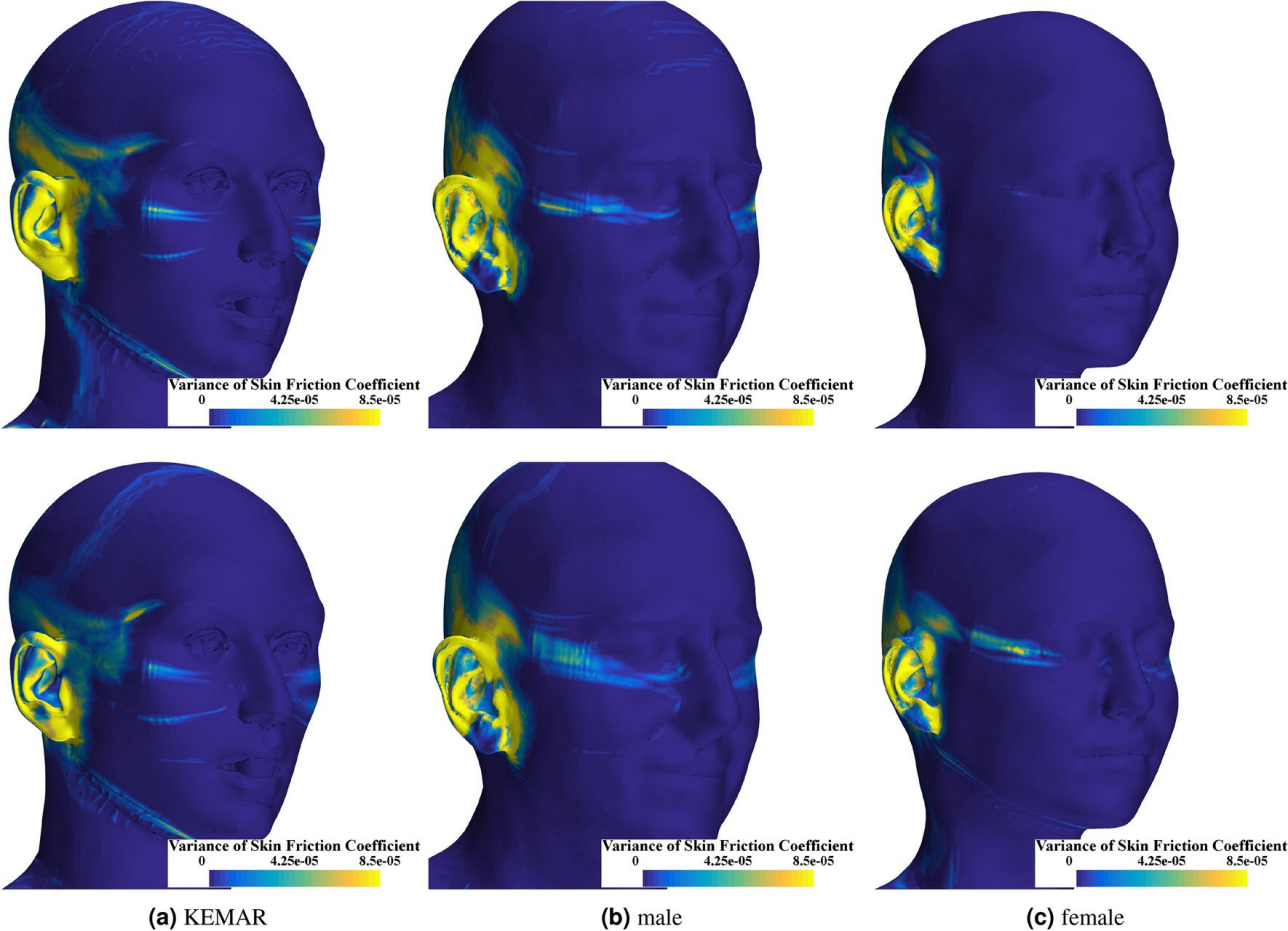
Variance of the skin friction coefficient $c_{f}$ for the three different head geometries at a wind velocity of 10 km/h (upper row) and 20 km/h (lower row).

Figure 8 shows the temporal variance of the skin friction coefficient for the three heads analyzed. A strongly fluctuating skin friction coefficient is correlated with the probability of flow separation and the formation of vortices. When comparing the three models, the geometric features of the head result in similar values for the skin friction coefficient variance, indicating the formation of vortices and flow separation bubbles at similar positions. In all three cases for the velocity of 10 km/h, a first maximum occurs in the area of the zygomatic bone, which spreads out in strips along the head geometry in the direction of flow. For the KEMAR, there is a further maximum in the area of the cheekbone. Especially in the area of the upper plane where the hearing aid is potentially located, the variance of the skin friction coefficient is strong for the three heads. In addition, the wall shear stress fluctuates very strongly for all three heads in the area of the entire pinna. The biggest difference when looking at the higher speed of 20 km/h is that the variation of the skin friction coefficient is emphasized for the two scanned heads, male and female, in the area of the lower jaw. Furthermore, at 20 km/h, an additional maximum occurs in the mouth area of the KEMAR and along the cheek for the male geometry.

**Fig. 9:**
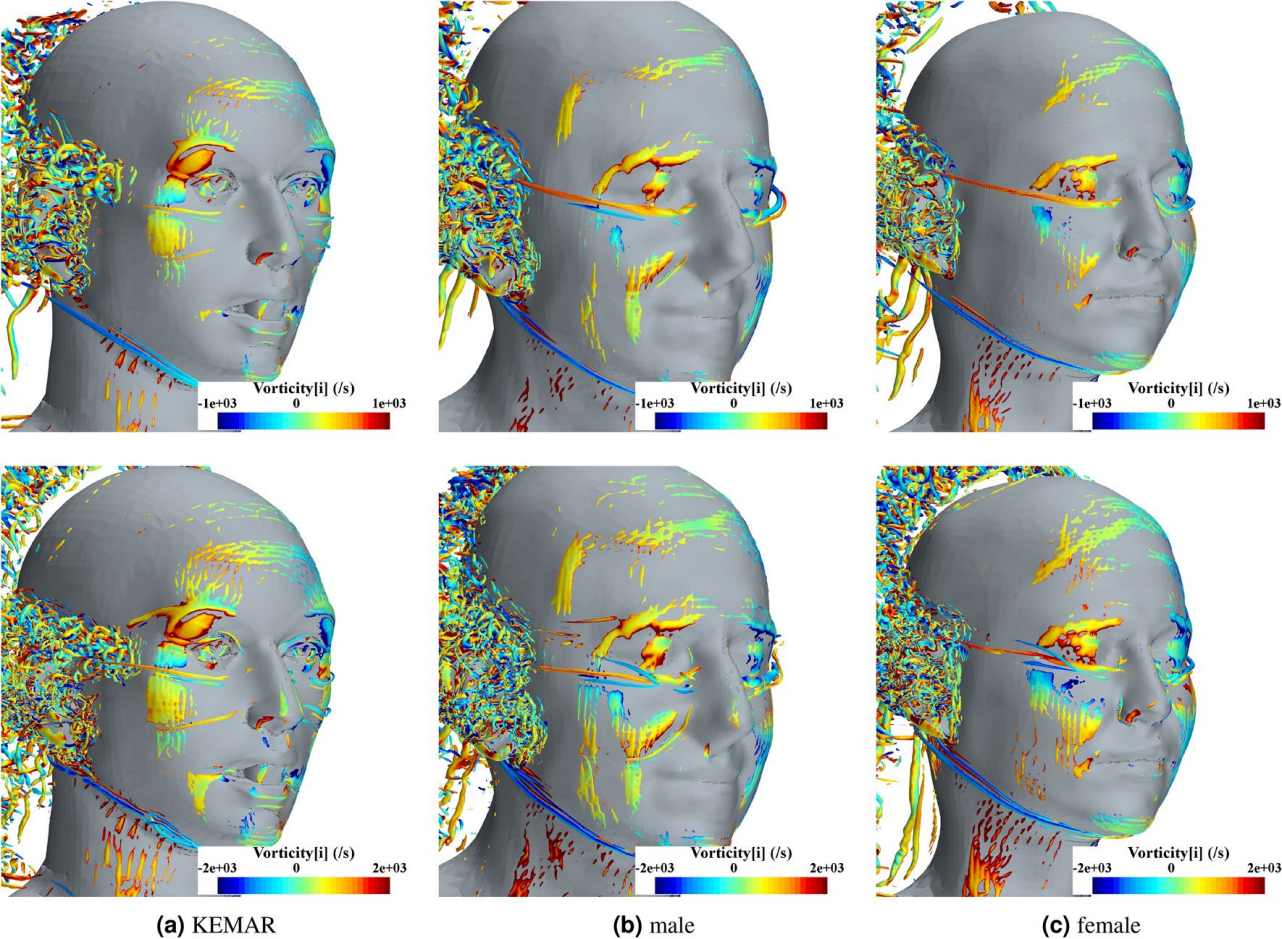
Visulization of the three-dimensional flow structures around the head geometries with the help of the $\lambda _{2}$-criterion for an Isovalue of $\lambda _{2}=-(50u_{\infty })^2/C_{head}^2$ at a representative timestep during simulation for a wind speed of 10 km/h (upper row) and 20 km/h (lower row). Where $C_{head}$ is the circumference of the head. The flow structures are additionally coloured with the vorticity around main flow direction (x-axis) to indicate the rotational direction of the vortices.

Figure 9 visualizes the formation of the vortices in the flow around the three heads at a representative time step during the simulation process. Isosurfaces of the $\lambda _{2}$-criterion are used, colored with the instantaneous vorticity around the x-axis to visualize the rotational direction of the vortices. The comparison of the three models shows that the vortices form similarly in the flow around a human head. For all three head geometries, an initial large-scale vortex detachment occurs in the area of the temple, which spreads in the upper plane towards the upper edge of the pinna. Another common feature of all three heads is the formation of a large vortex in the chin area, which spreads downstream along the lower jawbone. Shortly before the area of the pinna, small-scale vortex detachments occur in all three heads, extending over the entire ear geometry. Furthermore, small-scale vortices also form along the upper plane in the hearing aid area, which join the large vortices originating from the temple region. Finally, the isosurfaces of the $\lambda _{2}$-criterion are also strongly pronounced in the area just before the vertex for all three heads. The observation of the higher wind speed shows, in principle, a similar structure of vortex formation around the three head models. However, it is noticeable that the small-scale vortex shedding in the area of the upper edge of the pinna and in front of the pinna begins further upstream for all three heads at the higher wind speed. Furthermore, at 20 km/h, the flow also detaches at the trailing edge of the skull for all three head models. It is also interesting to note that the two large-scale vortices in the area of the temple and in the area of the lower jaw show an opposite direction of rotation in each of the configurations considered. For the wind speed of 20 km/h, videos of the visualization of the vortex dynamics are available in the supplementary material to this publication. Supplementary video 1, 2, and 3 shwo the vortex dynamics of the KEMAR, male and female head geometry.

### Wind pressure fluctuations at the skin and inside the ear

**Fig. 10:**
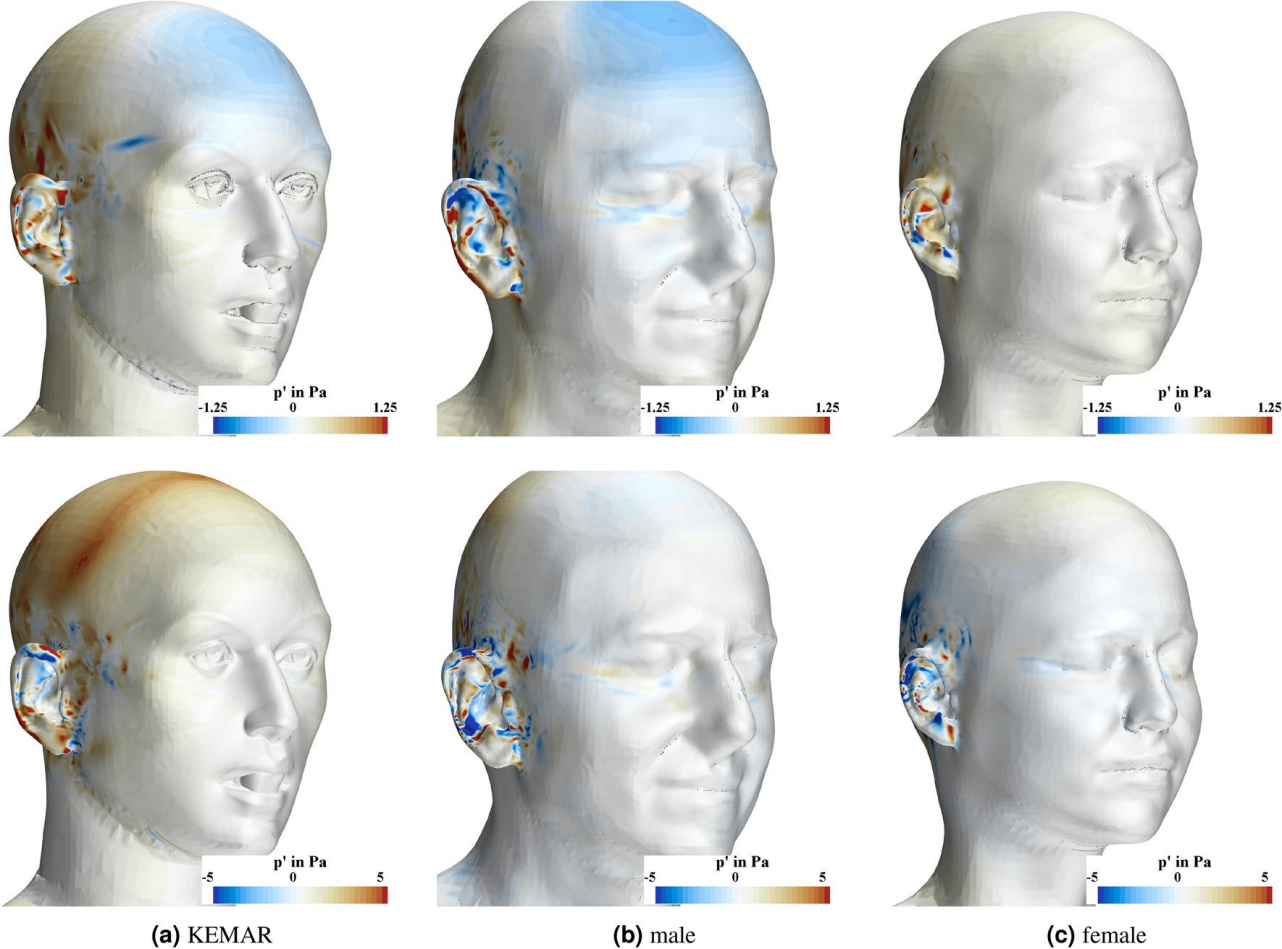
Visulization of the instantanous pressure fluctuations at a representative timestep during the simulation for the three head geometries at a wind speed of 10 km/h (upper row) and 20 km/h (lower row).

A snapshot of the temporal pressure fluctuations on the surface of the heads is visualized in Fig. 10. The temporal pressure fluctuations are calculated by subtracting the temporal mean of the static pressure from the static pressure at the current time step grid cell-wise. For all three head geometries considered, the pressure fluctuations are particularly pronounced in the area of the pinna and in the area above the pinna, where the hearing aid would be located. This illustration clearly indicates that the temporal pressure fluctuations in the area around the pinna are pronounced for all three head geometries and both wind velocities. To analyse the dominant frequency content of the time signal, a Fourier analysis of the pressure fluctuations is carried out at the monitoring points as defined in Fig. 4.

It is very well known that the human ear does not hear equally well at all frequencies. Especially at very low frequencies, which are the dominant frequencies in this matter, the perception threshold of the auditory system is greatly reduced. To take this into account, the A-weighting of technical noises is carried out in acoustics^40^. In a first step, the time signal of the calculated pressure is A-filtered. Then a power spectral density (PSD) is calculated from the filtered signal.

**Fig. 11:**
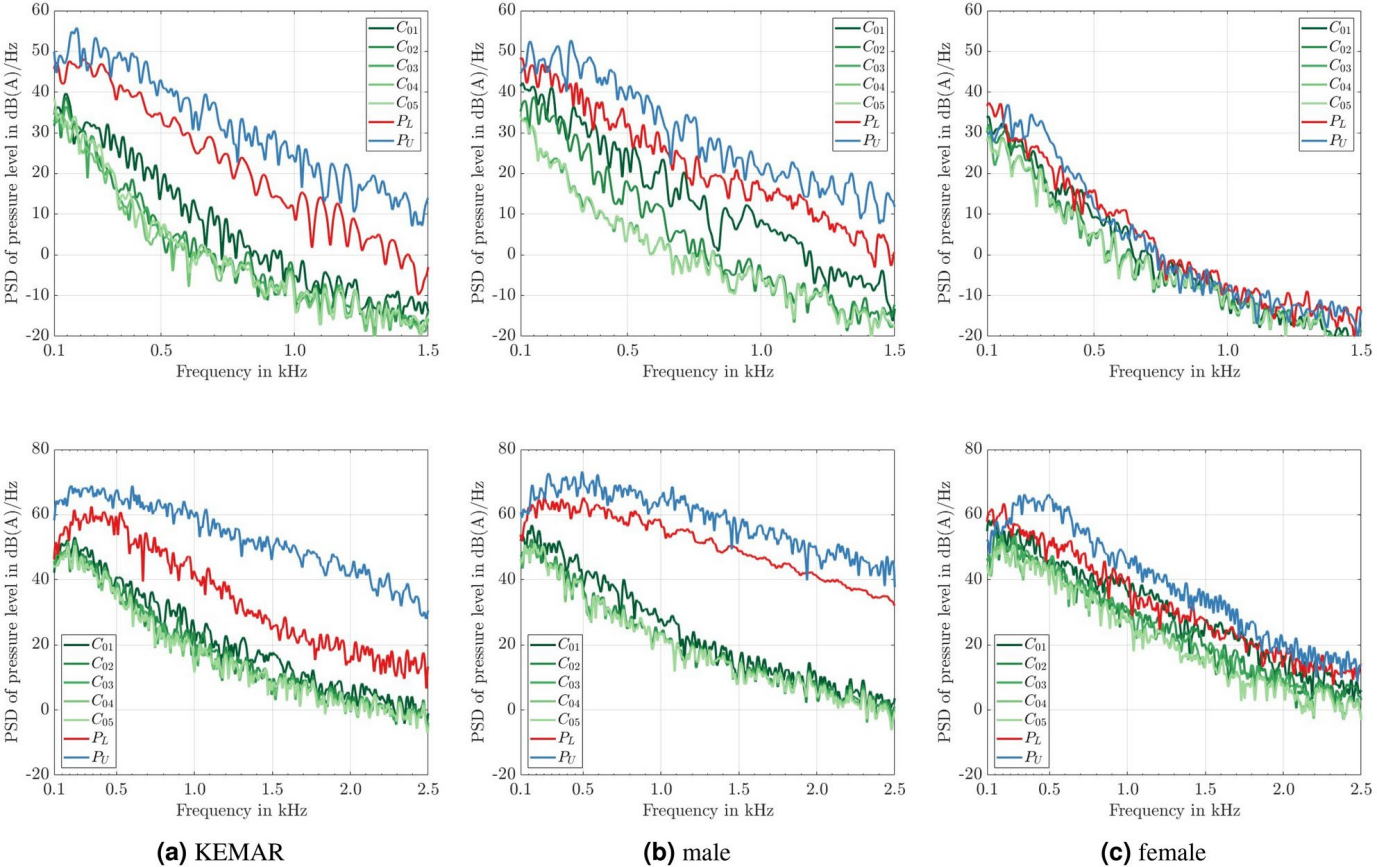
Plots of the A-weighted power spectral density (PSD) of the calculated pressure level at different monitor points for the three head models according to Fig. 4. Depicted for a wind speed of 10 km/h (upper row) and 20 km/h (lower row).

Figure 11 shows the spectra of the A-weighted pressure fluctuations as power spectral densities at selected monitor points for the three head geometries. The results for a wind velocity of 10 km/h are printed in the upper row, and the results for a wind velocity of 20 km/h are plotted in the lower row. According to the validation plot 6b the spectra here are shown in the frequency range between 100 Hz und 1500 Hz for the lower wind velocity and and between 100 Hz und 2500 Hz for the higher wind velocity.

The graph $P_{U}$, shown in blue, corresponds to the point in the upper plane and is, so to speak, the point at which the front microphone of a behind-the-ear hearing aid would be located. The graph $P_{L}$, plotted in red, is a point in the lower plane and is located at half the height of the ear canal but still outside the ear canal. An ITE device or a hearable would be approximately placed here and has the microphone recording the pressure signals at the position $P_{L}$. The points in different shades of green $C_{01}$ to $C_{05}$ are then located inside the ear canal, whereby these are each offset 2.5 mm further inside the ear canal, starting with $C_{01}$.

Firstly, as expected, the higher wind speed results in higher pressure levels. This is the case in the entire frequency range for all three head geometries. For the KEMAR and the male head geometry, the level difference in the range from 100 Hz to 1500 Hz is approximately 20 dB when increasing the wind speed from 10 km/h to 20 km/h. This is slightly more pronounced for the female head geometry (approximately 30 dB), which can be explained by comparing the vortical structures of Fig. 9c. Few vortical structures are visible at 10 km/h than in the case of 20 km/h.

For the KEMAR and the male head geometry, the level difference between $P_{U}$ and $P_{L}$ in the range from 100 Hz to 1500 Hz is approximately 10 dB. This tendency can be recovered for the female head geometry only in the case of 20 km/h, where the vortical structures are strong. Moreover, it is noticeable that for all three head geometries and both velocities, the pressure level at the potential hearing aid reference point $P_{U}$ reaches the highest levels over the entire frequency range. Point $P_{L}$ at the entrance outside the ear canal has the second highest level for all three cases and both wind speeds. The tendency is more pronounced for higher wind speeds. At this point, it is evident how poorly the position of the frontal microphones for BTE hearing aids is chosen from a fluid dynamical point of view.

When looking at the monitor points inside the ear canals, it becomes clear that for a wind speed of 10 km/h and 20 km/h there is a significant level reduction at the first point $C_{01}$ compared to $P_{L}$ for the KEMAR and the male geometry. This level reduction occurs between the entire frequency range. For the female head geometry, this reduction is way less noticeable. It occurs between 100 Hz and 700 Hz for both velocities. A decrease in level between $C_{01}$ and $C_{02}$ is observable for all considered head geometries and wind speeds. In particular, a strong reduction of the pressure levels is visible for the KEMAR at 10 km/h (300 Hz to 1000 Hz) and for the male head geometry from 100 Hz to 1500 Hz. From point $C_{03}$ onwards, the spectra of the pressure levels for these head geometries are almost identical. Only for the male head geometry, there is still a reduction in the pressure level between points $C_{02}$ and $C_{03}$ in the range between 100 Hz and 800 Hz (at 10 km/h). In all variants, there is no significant reduction in the pressure level from point $C_{03}$ onwards, which corresponds to a microphone position at a depth of penetration into the ear canal of 7.5 mm.

### Behind the ear devices are exposed to the highest wind pressure fluctuations

To obtain an A-weighted total sound pressure level $L_{pA}$, the levels of the individual frequency points (e.g. see Fig. 11) considered are added energetically afterwards (see Tab. 1). In this case and based on the validation, the level values in the frequency range between 100 Hz and 1500 Hz for 10 km/h and between 100 Hz and 2500 Hz were taken into account in the energetic addition.

**Table 1:** A-weighted overall pressure levels $L_{pA}$ for the spectra from Fig. 11, considering the frequency range from 100 Hz to 2500 Hz for both wind velocities.

		KEMAR	male	female
10 km/h	$P_{U}$	75.61 dB(A)	73.60 dB(A)	56.09 dB(A)
	$P_{L}$	70.12 dB(A)	68.10 dB(A)	55.51 dB(A)
	$C_{05}$	54.21 dB(A)	50.89 dB(A)	47.77 dB(A)
20 km/h	$P_{U}$	94.84 dB(A)	94.84 dB(A)	89.91 dB(A)
	$P_{L}$	85.35 dB(A)	91.20 dB(A)	83.95 dB(A)
	$C_{05}$	70.98 dB(A)	72.10 dB(A)	72.80 dB(A)

The corresponding results for the A-weighted total sound pressure levels of the signals of Fig. 11 are shown in table 1. By calculating the overall level, it is also possible to compare the different head geometries and monitor points with each other. Firstly, it is noticeable that the highest pressure levels achieved for both flow velocities are obtained for the KEMAR and the male head geometry at $P_{U}$. For each configuration, the pressure levels at $P_{U}$ are higher compared to $P_{L}$, which is higher than the pressure levels at $C_{05}$. For a speed of 20 km/h, a pressure level value 20 dB(A) higher than for a speed of 10 km/h is achieved at $P_U$ in these cases. For the female head, the level difference at point $P_{U}$ between the two speeds is even more than 30 dB(A). A similar increase is detectable for the other pressure monitoring points. The attenuation of the pressure level from $P_U$ into the ear canal $C_{05}$ is similar when changing the wind speed. For the KEMAR, 19.4 dB(A) at 10 km/h and 23.8 dB(A) at 20 km/h. 22.7 dB(A) at 10 km/h and 20 km/h for the male head geometry. Likewise, for the female head geometry, 8.3 dB(A) at 10 km/h and 17.1 dB(A) at 20 km/h. It seems to depend mostly on the head geometry and the flow pattern. In particular, the flow pattern of the female head geometry changed when transitioning from 10 km/h to 20 km/h (see Fig. 9c).

For the flow velocity of 20 km/h, the differences inside the ear canal between the three heads are less pronounced, which is in line with the findings of Fortune and Preves^4^. With a value of 72.80 dB(A), the female head achieves the highest value, followed by the male head geometry with 72.10 dB(A) and the KEMAR with 70.98 dB(A). For a better sense of the values determined for the overall sound pressure level in dB(A), a few examples of typical everyday sounds are given below. For example, the average sound pressure level in a library is around 40 dB(A), in a busy office it can be measured at an average of 60 dB(A), and 80 dB(A) corresponds to the value measured on a busy road. Finally, 100 dB(A) can be measured in the vicinity of a pneumatic hammer^41^. At 20 km/h, the pressure levels recorded outside the ear from wind noise are louder than the measured values on a busy road.

### Anatomically exact ear canals most strongly dampen turbulent velocity fluctuations

Next, the turbulent velocity fluctuations based on the turbulent kinetic energy are analyzed for the defined monitor points inside the auditory canal.

**Fig. 12:**
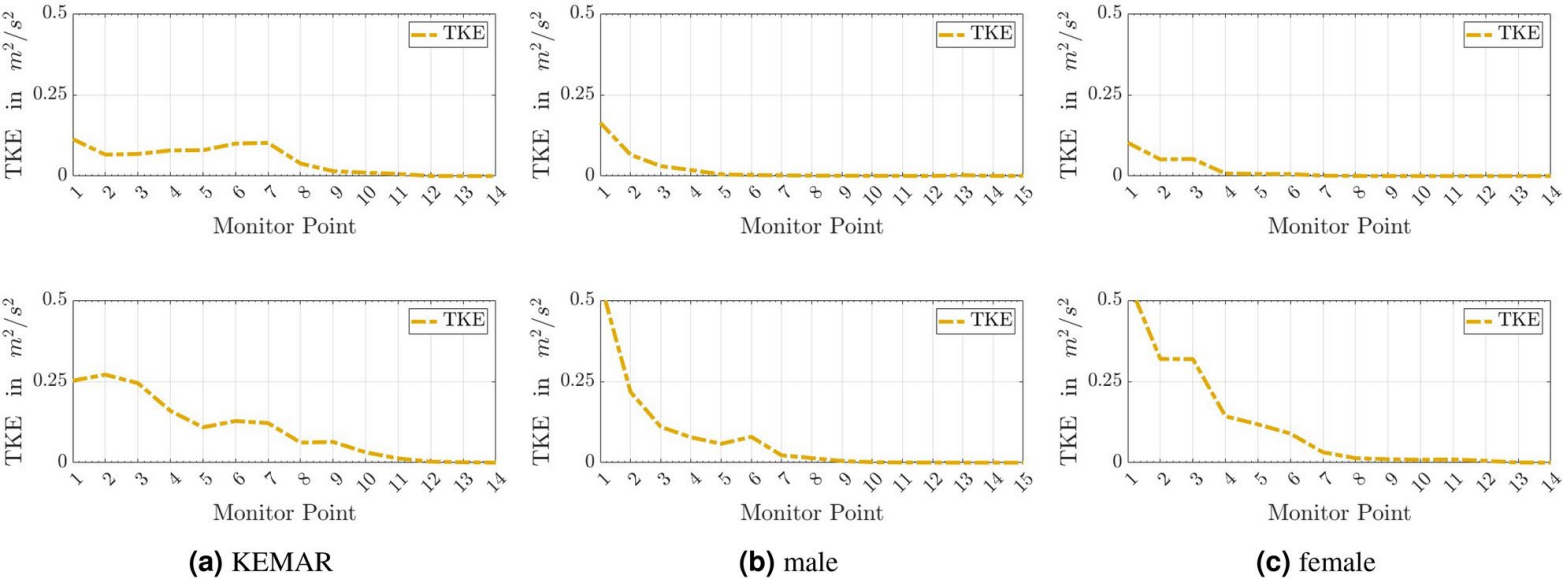
Plots of the turbulent kinetic energy (TKE) for the monitor points within the ear canal of the head models. For the inflow velocities of 10 km/h (upper row) and 20 km/h (lower row).

In Fig. 12 we analyze the time averaged turbulent kinetic energy (TKE), being the sum of the diagonal elements of the Reynolds stress tensor.The results for a wind velocity of 10 km/h are shown in the upper row, while in the lower row, the graphs for a wind velocity of 20 km/h are depicted. Figure 12 shows that at a wind speed of 10 km/h, the turbulent fluctuations for the two physiological ear canals of the male and the female are already strongly damped at point $C_{04}$. From point $C_{05}$, the turbulent kinetic energy follows the zero line in both cases. In contrast, the fluctuations in the ear canal of the KEMAR remain at a constant level up to the point $C_{07}$. The zero line is not reached until point $C_{11}$.

For the flow velocity of 20 km/h, it is found that the initial values of the turbulent kinetic energy when entering the auditory canal are increased for all models compared to the lower wind speed. The scaling is proportional to $u_\infty ^2$. For the two natural auditory canals, there is again a strong attenuation of the velocity fluctuations in the direction of the eardrum. From point $C_{08}$, the zero line is almost reached for both real head geometries from the IHA database. A different picture emerges for the artificially elongated auditory canal of the KEMAR. The sharpest drop in velocity fluctuations starts at point $C_{03}$ until $C_{05}$, then a slight increase until point $C_{07}$ (the end of the physiological section and the start of the cylindrically shaped extension) occurs. The turbulent kinetic energy is completely attenuated at point $C_{12}$. To summarize, it can be deduced from the results that the auditory canals, which are anatomically precisely modeled over their entire length, dampen the turbulent velocity fluctuations more effectively than it is the case with the KEMAR’s auditory canal extended with a cylinder. The slight increase in the TKE for the KEMAR (between $C_{05}$ and $C_{08}$) and the male head geometry (between $C_{05}$ and $C_{07}$) at 20 km/h is due to recirculations inside the ear cavity.

## Discussion and conclusions

In this work, a first study of the inherently three-dimensional flow field around the human head in two everyday wind noise situations such as jogging or cycling with the help of computational fluid dynamics has been performed. Previous experimental investigations were limited to the qualitative visualization of the flow field with smoke experiments^42^, the recording of the three components of the flow velocity independently of each other using Laser Doppler Anemometry^5^ or the representation of the flow field in a plane (two-dimensional) using Particle Image Velocimetry^43^. The quantitative measurement of coherent three-dimensional structures close to highly curved boundaries is very challenging for classical Particle Image Velocimetry^44,45^ or impossible inside the human ear canal. As the boundary layer has to be recorded in the range of $y^{+}$ =1, the human head surface has a complex three-dimensional curvature and flow data inside the ear canal is essential, validated flow simulations are used to study the flow around the human head. Firstly, the numerical approach was validated with the help of a wind tunnel experiment. It was shown that the pressure fluctuations measured in the experiment on the eardrum of the generic artificial head KEMAR 45BB-10 can be reproduced well by wallresolved large eddy simulations (LES) for results in the frequency range between 100 and 2500 Hz and wind speeds of 10 km/h and 20 km/h.

Building upon the validated numerical model, the simulated flow field of three different head geometries (KEMAR, female, male) was compared. The geometric models of a male and a female upper body were taken from the IHA database for acoustic research^21^. The qualitative comparison of the simulation results of the three heads showed dominant universal flow features. The observed characteristics around the human body are stagnation in front of the body in wind approach direction, the flow around the head and chest, and flow detachment around the sides, the side of the head, the shoulders, and the top of the head. All these flow phenomena, the ear wake, the wake around the head, and over the shoulder interact with each other. In the area of the zygomatic bone at the height of the temple, a large-scale vortex formation occurs for all three heads and both wind velocities. It then spreads downstream towards the upper edge of the pinna where a Behind-The-Ear hearing aid is typically located. There, the flow strongly interacts with the ear and potentially with the Behind-The-Ear device, which causes these strong wind sound levels. These flow interactions can be considered in an informed design process of structures to reduce wind-induced noise^46,47^. The visualizations of the flow structures indicate where recording microphone positions should be ideally placed in hearing aids and hearables to be the least exposed to wind loading.

A few centimeters upstream of the upper edge of the pinna, flow separation leads to the formation of many small-scale vortices, which are likewise convected towards the location of the hearing aid. At the height of the ear canal flow separation and the associated formation of vortices occur directly in front of the pinna. With increasing wind speed, the formation of the small-scale vortices shifts upstream. The simulation results indicate that a strong variance of the skin friction coefficient and a strongly negative value of the pressure coefficient are indicators of vortex separation. An evaluation of the time-dependent pressure fluctuations on the head surface of the three models at a representative timestep showed that these are particularly pronounced in the area of the pinna and at the upper edge of the pinna.

A quantitative evaluation of the simulation results was conducted at multiple result evaluation points. These points were placed at typical microphone positions for hearing devices on the one hand ($P_{U}$, $P_{L}$), and equidistantly in the ear canal on the other hand ($C_{01}-C_{15}$). The evaluation of the power spectral density of the sound pressure showed that for all three head geometries and both wind velocities, the pressure level at the location of the potential Behind-The-Ear hearing aid (BTE) is greater over the entire frequency range than at the entrance to the ear canal, where a hearable or an In the ear device (ITE) would be located.

For the wind velocity of 10 km/h, the wind noise levels are pronounced in the low-frequency range, then fall steeply for a certain frequency range until the curve crosses the 0 dB line and then remain almost constant at levels below 0 dB. Generally, higher overall A-weighted pressure levels were calculated for the male head model and the KEMAR compared to the female head model. It can be explained by the fact that the male geometry and the KEMAR have a larger head circumference than the female geometry. As a result of the larger head circumference, the length for developing the turbulent boundary layer between the flow stagnation in the nose area until reaching the hearing aid position is the longest.

An investigation of the turbulent kinetic energy inside the different ear canals was carried out next. The comparison of the fluctuations inside the auditory canals revealed that the anatomically correctly represented auditory canals reduce the velocity fluctuations more effectively than the artificial canal of the KEMAR. This conclusion holds for both wind speeds investigated.

The results indicate that the flow field for frontal flow on the human head can be generalized to a large extent. Irrespective of the exact head geometry, vortex shedding always occurs in the temple area, shortly upstream of the upper edge of the pinna and over the entire area of the pinna. Nature has designed the shape of the tragus on the pinna and the geometric tapering of the ear canal to suppress wind noise during natural hearing optimally. When using hearing aids or hearables, these advantages of wind noise suppression can only be utilized partially or not at all. The position in which the front microphones of current Behind The Ear hearing aids are located is probably the least favorable on the entire head from a purely wind-related perspective.

With the interpretation of the quantitative results presented here, it can be explained from a fluid mechanics perspective why Behind The Ear hearing aids always perform worse in the wind than In The Ear hearing aids as stated by Dillon^5^ and Zakis^6^. The latter, in turn, perform worse than In The Canal devices, which is consistent with the results of Fortune and Preves^4^. As a rule of thumb for wind-noise aware design, a modern hearing aid or hearable should be placed recessed in the ear canal wherever possible.

In conclusion, for reasons of reproducibility and simulation capability, the tests carried out here are limited to people without head hair. It can be assumed that the wind noise problem in the area of the upper edge of the pinna is significantly attenuated by head hair, the wearing of bonnets, or a pronounced beard. Furthermore, only wind noise due to purely hydrodynamic pressure fluctuations was considered in this study. Flow-acoustic noises resulting from the wind may play a role primarily within the ear canal and will be investigated in a subsequent study.

The objective of future work is to combine the existing simulation model with aeroacoustic methods^48^ for real-life outdoor communication situations. The future work will provide new physical insights for improving virtual reality realistically and supporting future design and development of communication and medical devices for optimal performance and user satisfaction.

## Supplementary Information

Supplementary Information 1.

Supplementary Information 2.

Supplementary Information 3.

Supplementary Information 4.

## Data Availability

The numerical and experimental data underlying this study are available from the corresponding author upon reasonable request. Any data connected to the two physiological heads is restricted and consent must be obtained from the respective parties.
