# Supplementary material for: Investigation of the physical driving mechanisms of wind noise in hearing devices by computational fluid dynamics
Source: Sci Rep. 2025 Mar 25;15:10290. doi: 10.1038/s41598-025-93303-y (PMC11937389; doi:10.1038/s41598-025-93303-y)
Supplement: Supplementary file 1 — Supplementary Information 1. [file 41598_2025_93303_MOESM1_ESM.doc]

**Supplementary Information Guide**

The manuscript titled “**Investigation of the physical driving mechanisms of wind noise in hearing devices by computational fluid dynamics**” prepared by Riedel et al. has three supplementary video files. Titles for each file and summaries for each file are described below.

1. Titles for each file:
   **Supplementary Video 1:** Visualisation of the vortical structures around the artificial head KEMAR at 20 km/h wind speed (KEMAR_lambda2_vorticity_visualization_20_kmh.mp4)

**Supplementary Video 2:** Visualisation of the vortical structures around a male head at 20 km/h wind speed (MALE_lambda2_vorticity_visualization_20_kmh.mp4)

**Supplementary Video 3:** Visualisation of the vortical structures around a female head at 20 km/h wind speed (FEMALE_lambda2_vorticity_visualization_20_kmh.mp4)

1. Summary for each file
   **Supplementary Video 1:**

The video shows the vortex dynamics at the Kemar artificial head in a frontal flow at a wind speed of 20 km/h. The flow structures are shown with the help of the λ_2-_criterion and are shown in the colour of the vorticity in the main flow direction.

**Supplementary Video 2:**

The video shows the vortex dynamics at a male head in a frontal flow at a wind speed of 20 km/h. The flow structures are shown with the help of the λ_2-_criterion and are shown in the colour of the vorticity in the main flow direction.

**Supplementary Video 3:**

The video shows the vortex dynamics at a female head in a frontal flow at a wind speed of 20 km/h. The flow structures are shown with the help of the λ_2-_criterion and are shown in the colour of the vorticity in the main flow direction.
